# Biosecurity Competencies and Educational Needs Among Veterinary Professionals: A Multinational Cross‐Sectional Survey

**DOI:** 10.1155/tbed/7275694

**Published:** 2026-09-15

**Authors:** Claude Saegerman, Véronique Renault, Constance Wielick, Blerta Mehmedi, Jorge Ron-Román, Amel Adel, Bachir Souley-Kouato, A. K. M. Anisur Rahman, Marie-France Humblet, Marion Allano

**Affiliations:** ^1^ Research Unit in Epidemiology, Risk Analysis and Biosecurity Applied to Veterinary Sciences (UREAR-ULiege), Fundamental and Applied Research for Animals and Health (FARAH) Center, Faculty of Veterinary Medicine, University of Liege, Liege, Belgium, ulg.ac.be; ^2^ Council Member of the World Animal Biosecurity Association (WABA), Belgium; ^3^ Regional Association for Animal Registration and Health (ARSIA) asbl, Ciney, Belgium; ^4^ Department of Veterinary Medicine, Faculty of Agriculture and Veterinary, University of Prishtina, Prishtina, Kosovo, uni-pr.edu; ^5^ Laboratorio de Mejoramiento Genético y Sanidad Animal, Grupo de Investigación en Sanidad Animal y Humana (GISAH), Departamento de Ciencias de la Vida y de la Agricultura, Universidad de las Fuerzas Armadas ESPE, Sangolquí, Ecuador, espe.edu.ec; ^6^ Institute of Veterinary Sciences, University of Blida 1, Blida, Algeria, univ-blida.dz; ^7^ Institut National de la Recherche Agronomique du Niger (INRAN), Niamey, Niger; ^8^ Epidemiology and Preventive Medicine Laboratory, Department of Medicine, Bangladesh Agricultural University, Mymensingh, Bangladesh, bau.edu.bd; ^9^ Department of Occupational Protection and Hygiene, Unit Biosafety, Biosecurity and Environmental Permits, University of Liège, Liège, Belgium, ulg.ac.be; ^10^ Faculty of Veterinary Medicine, Université de Montréal, Saint-Hyacinthe, Canada, umontreal.ca

**Keywords:** animal biosecurity, competency assessment, educational needs, multinational survey, One Health, practitioners, veterinary education

## Abstract

The importance of animal biosecurity is growing, and it has been incorporated into the veterinary medicine curriculum, as well as into the European Animal Health Law and the World Organization for Animal Health (WOAH) Terrestrial Animal Health Code. However, few studies have been conducted to date on the self‐perceived competencies acquired and the educational needs of veterinary students and practitioners in animal biosecurity. To address this, an online survey involving 651 participants from 66 countries across 5 continents was conducted (with predominance of Europe). The three sections of the survey were participant profiles, perceptions of their competencies, and additional training needs. The survey participants work in veterinary clinics, ambulatory practices, and academic environments. Main animal species managed were ruminants, companion animals, equids, poultry, and pigs. The level of agreement regarding the competencies acquired in relation to the managed species was consistent, except for the biosecurity audits. Competencies increased markedly between graduation and the tenth year of practice, followed by a slower progression. A total of 276 participants expressed a strong demand for training in biosecurity but a lower need for cleaning and disinfection protocols. No significant intercontinental differences (Africa, America, and Europe) were found for competencies in risk analysis and biosecurity audits. However, Africa showed a significantly lower median priority score for competencies related to cleaning/disinfection, animal isolation, and information sourcing. Identifying priority competencies and training gaps will enable better‐targeted educational interventions, thereby contributing to Sustainable Development Goals 3 (good health and well‐being) and 10 (reduced inequalities).

## 1. Introduction

**Figure 1:**
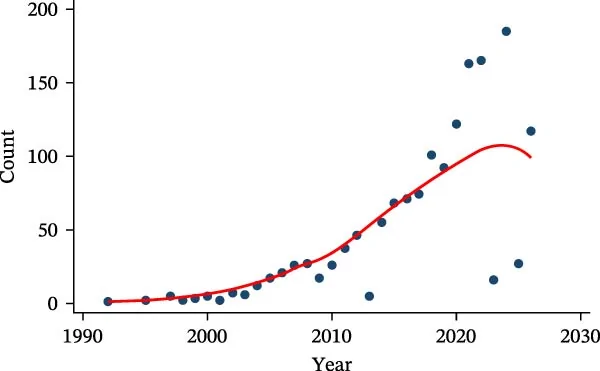
Number of PubMed (US National Library of Medicine, National Institutes of Health) publications (vertical axis) on May 10, 2026, that mention “biosecurity” and “animal” in their title or abstract (points are observed data; the red solid line was obtained using the Lowess smoothing method [1]).

The importance of animal biosecurity is increasing globally (Figure 1). Animal biosecurity has been progressively integrated into the veterinary medicine curriculum [2, 3] as well as in the European Animal Health Law [4, 5]. In terms of education, biosecurity was recently integrated by the World Health Organization (WHO), Food and Agriculture Organization, and World Organization for Animal Health (WOAH) as a competency into the One Health field epidemiology framework, particularly in domain number six, which relates to infection prevention and control, biosafety, and biosecurity [6]; and it includes three subdomains, that is, preparedness, implementation procedures, and evaluation of continuous quality improvement [6]. In addition, a new chapter of the Terrestrial Animal Health Code on biosecurity is currently being prepared by the WOAH, and the World Animal Biosecurity Association (WABA) was recently created [5].

Despite this growing importance, few surveys have been conducted on biosecurity education, competencies, and training needs; these studies were frequently generic and only related to one or a few countries [7, 8]. Mehmedi et al. [8] recommends a framework for tailored biosecurity training of veterinarians, comprising five progressive modules: introduction, behavior change and communication, disease transmission and risk assessment, emergency response and clinical biosecurity, and on‐farm practices. For veterinary practitioners, a mixed approach, including a theoretical and a practical part involving the creation of a biosecurity plan and the writing of a follow‐up report, was the most important training feature [7]. Also, for veterinary students, building a solid understanding of biosecurity main principles was identified as an essential component of training [7].

However, in view of these developments, it would be very useful to understand the perception of species‐related biosecurity competencies and to identify further training needs for final‐year veterinary students and veterinary practitioners. A questionnaire survey aiming to address this issue is proposed. Identifying biosecurity competencies and needs will enable the development of evidence‐based future training.

## 2. Materials and Methods

### 2.1. Data Privacy and Ethical Committee

A cover letter and a questionnaire were submitted to and approved by the data privacy officer of the University of Liège to verify compliance with the General Data Protection Regulation. The survey was then approved by the Ethics Committee for the Humanities and Social Sciences at the University of Liège (Reference Number 2025–50).

### 2.2. Definition

This survey focuses more on biosecurity competencies than on skills [9]. Skills can be defined as the ability to apply knowledge and to use know‐how to complete tasks and solve useful problems, usually in the workplace. Competencies, on the other hand, can be defined as broader attributes that refer to the ability to apply knowledge and social and/or methodological abilities in work, study, professional, and personal development situations. Competency is not limited to the cognitive area but also encompasses functional/technical areas, as well as interpersonal competencies and values.

### 2.3. Questionnaire Survey

An online questionnaire survey (in French, English, and Spanish) was conducted using Kobo [10]. The questionnaire included a brief cover letter and was divided into three sections: the participant’s profile, their perception regarding the level of confidence in the biosecurity competencies, and the needs for further training (Supporting Information 1: Questionnaire S1).

The questionnaire survey was distributed across multiple continents by first, through the WABA (https://wababiosecurity.com/), and second, through a snowball sampling strategy, that is, the first wave of respondents distributed the questionnaire link to others via e‐mail [10].

### 2.4. Statistical Analysis

The perceived level of agreement for each competency relative to animal species managed by the participants was assessed using a five‐point Likert scale (disagree [D], partially disagree [PD], neutral [N], partially agree [PA], and agree [A]), plus an additional option, that is “does not apply to my practice.”

To rank the competencies in order of decreasing priority, the following competency ratio was generated:

(1) Competency ratio=number of participants who selected PD and Dnumber of participants who selected PA and A,

With PA, A, PD, and D being, respectively, the number of participants that PA, agree, PD, and disagree that they have the necessary competency.

A ratio close to zero indicates that the subject area is adequately covered, whereas an increasing deviation from zero reflects a need for improvement in that area.

Cluster analysis was carried out using a regression tree analysis (Salford Predictive Modeler, Version 8.2, Salford Systems, San Diego, CA, USA) [11]. Because the competency ratio is a continuous variable, the aim was to obtain groups of competencies characterized by minimal within‐group variance and comparable relative importance.

In addition, the statistical differences between median values after clustering the competencies were assessed using a nonparametric Kruskal–Wallis equality‐of‐populations rank test followed by an ordered regression analysis.

### 2.5. Sensitivity Analysis

The competence ratio (Equation 1) did not account for the neutral responses. Neutral respondents are those who are uncertain or lack a definitive opinion on their competence. If half of these respondents (50%) can become with agreement (“agree” or “partially agree”) or disagreement (“disagree” or “partially disagree”) of their competence, this scenario have strictly no effect on the competence ratio. However, if this percentage changes, the competence ratio can be affected. To test if these respondents have an important effect on the competence ratio calculated, a sensitivity analysis was performed. First, we considered all the current 30 ratios and attributed a rank to each of those (i.e., reference scenario). Second, we have simulated two additional alternative scenarios. In the first scenario, we expected that 40% of neutral would become with agreement and 60% would become with disagreement. In the second scenario, we expected that 60% of neutral would become with disagreement and 40% of neutral would become with agreement. For these two additional scenarios, we attributed a rank to each ratio recalculated. Third, we compared the two rankings of the two alternative scenarios with the ranking of the reference scenario and counted how many ratios were affected by more than +/− 3 ranks (10% of the total number of ratio).

## 3. Results

### 3.1. Description of Participants

This survey included 651 participants from 66 countries across 5 continents (with a predominance of Europe) (Table 1). The participants are final‐year veterinary students (*N* = 135) and veterinary practitioners who graduated, respectively, less than 5 years ago (*N* = 85), between 5 and 10 years ago (*N* = 63), and more than 10 years ago (*N* = 309). In addition, 59 respondents contributed to the survey, including academics, researchers, veterinary inspectors, laboratory‐based veterinarians, and agronomists involved in animal production.

**Table 1:** Profile of veterinary students and practitioners who participated in a cross‐sectional survey about biosecurity practices, 2026.

Participants (%)	Africa	America	Asia	Europe	Oceania	Total
Final‐year veterinary students	20 (17.9)	51 (48.6)	4 (20)	60 (14.6)	—	135 (20.7)
Veterinary practitioners graduated less than 5 years ago	29 (25.9)	5 (4.8)	—	51 (12.4)	—	85 (13.1)
Veterinary practitioners graduated between 5 and 10 years ago	17 (15.2)	7 (6.7)	2 (10)	37 (9.0)	—	63 (9.7)
Veterinary practitioners graduated more than 10 years ago	35 (31.25)	26 (24.8)	3 (15)	243 (59.0)	2 (100)	309 (47.5)
Others	11 (9.8)	16 (15.2)	11 (55)	21 (5.1)	—	59 (9.1)
Total of participants	112 (17.2)	105 (16.1)	20 (3.1)	412 (63.3)	2 (0.3)	651 (100)
Total of countries	22 (33.3)	8 (12.1)	9 (13.6)	25 (37.9)	2 (3.0)	66 (100)

The participants of the survey worked in veterinary clinics (*N* = 305; 46.9%) or in ambulatory practices (*N* = 121; 18.6%), in academic environments (*N* = 173; 26.6%), in public or private associations, cooperatives, or organizations (*N* = 61; 9.4%), in governmental bodies (*N* = 61; 9.4%), in the pharmaceutical industry (*N* = 11; 1.7%), or others (*N* = 67; 10.3%). Among all participants, 526 (80.8%), 107 (16.4%), 13 (2%), and 5 (0.8%) work for one, two, three, or four different structures. For participants working in two settings, the most frequent combinations were veterinary clinic and ambulatory practice (*N* = 36; 33.6%) and veterinary clinic and academic environment (*N* = 29; 27.1%).

**Figure 2:**
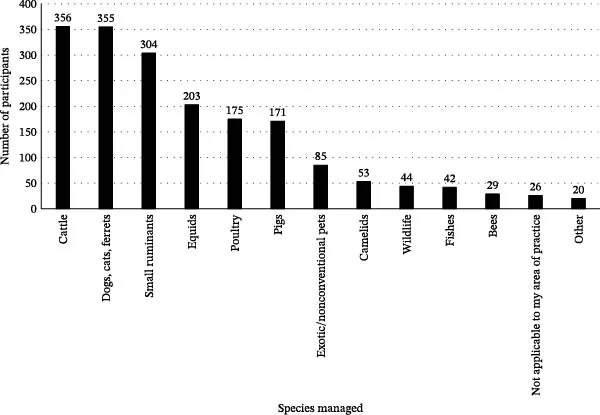
Distribution of the species managed by veterinary students and practitioners who participated in a cross‐sectional survey about biosecurity practices, 2026. One participant can manage several species.

The participants managed one or more species (Figure 2). Regression tree analysis revealed that six species were statistically more prevalent (Supporting Information 2: Figure S1), that is, cattle, pets (dogs, cats, and ferrets), small ruminants, equids, poultry, and swine.

### 3.2. Level of Agreement Regarding Perceived Biosecurity Competencies With the Species Managed

Participants were asked to self‐evaluate their perceived biosecurity competencies in relation to the species they managed, as listed in Supporting Information 3: Table S1. A general trend was observed. Agreement levels regarding the competencies acquired for species managed were generally consistent (8.9%–16.7% disagree or PD; 9%–12.2% neutral; 71.1%–81.2% PA or agree), with the exception of biosecurity audits, for which the distribution was 21.9%, 15.5%, and 62.6%, respectively. Each biosecurity competency in Supporting Information 3: Table S1 is quantified as the ratio of respondents expressing disagreement (partial or full) to those expressing agreement (partial or full). The ratios span from 0.05, reflecting strong overall agreement, to 0.91, where agreement only marginally exceeds disagreement, signaling notable improvement needs.

**Figure 3:**
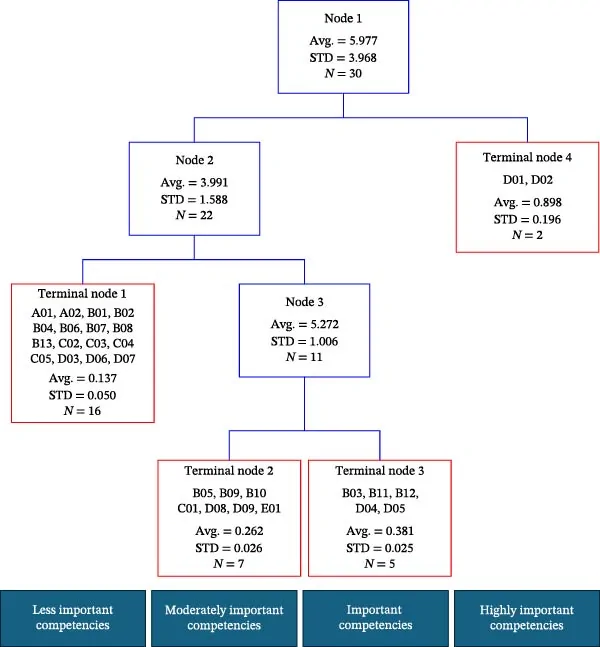
Groups of biosecurity competencies identified through a regression‐tree analysis by veterinary students and practitioners who participated a in a cross‐sectional survey about biosecurity practices, 2026. Avg, average; STD, standard deviation; *N*, number; A01 and A02, competencies associated with cleaning and disinfection protocols, respectively, for surfaces (A01) and for equipment (A02); B01–B13, competencies related to the isolation of animal suspected of having a contagious and/or zoonotic disease in adapting a facility within a veterinary hospital or clinic (B01), in advising the owner on how to isolate, respectively, a pet (B02), an exotic/nonconventional pet (B03), a ruminant (B04), a camelid (B05), a pig (B06), a poultry (B07), a horse (B08), an animal in a dog breeding facility (B09), an animal in a kennel, a cattery, a boarding kennel or a shelter for dogs/cats (B10), a batch of animals in a fish farm (B11), a colony in an apiary (B12), and a shelter/wildlife rehabilitation center (B13); C01–C05, competencies related to risk analysis, respectively, to carry out an analysis of infectious risks relevant to the field of practice (C01), to design procedures aimed at reducing the risk of contagion between patients within a veterinary clinic (C02), to design protocols intended to protect both myself and my staff from exposure to zoonotic pathogens (C03), to communicate with owners regarding the potential hazards linked to contagious and/or zoonotic diseases affecting their animals (C04), and to advise animal owners on strategies to mitigate the risks associated with contagious and/or zoonotic diseases affecting their animals (C05); D01–D09, competencies related to biosecurity audits, respectively, in a community of dogs or cats, or in a ferret‐breeding facility (D01), at a seller of exotic/nonconventional pets (e.g., reptiles, exotic birds) (D02), in a ruminant husbandry (D03), in a camelid husbandry (D04), in a horse farm (D05), in a pig farm (D06), in a poultry farm (D07), in a fish farm (D08), and in an apiary (D09); and E01, competency related to reference information (E01).

To determine the relative importance of biosecurity competencies requiring improvement, regression tree analysis was applied to all ratios. Four distinct groups of biosecurity competencies were identified (Figure 3), three of which exhibited a mean ratio greater than 0.25. Most competencies included in these three groups are related to animal isolation procedures (thematic area B) and biosecurity audits (thematic area D).

The first group of competencies (terminal node 4) displayed a mean ratio close to 0.9, indicating substantial room for improvement. This group includes two audit‐related competencies, that is, conducting a biosecurity audit in a community of dogs or cats or on a ferret farm (D01) and conducting a biosecurity audit at a retailer of exotic/nonconventional pets (e.g., reptiles, exotic birds) (D02).

The second group of competencies exhibited an average ratio of 0.38, suggesting that over one‐third of participants reported insufficient confidence in these competencies. This group includes five competencies related to animal isolation practices and biosecurity auditing: advising owners on how to isolate exotic/nonconventional pets (e.g., reptile, bird, small mammal) suspected of having a contagious and/or zoonotic disease (B03), on how to isolate a group of animals suspected of having a contagious and/or zoonotic disease in a fish farm (B11), on how to isolate a colony suspected of having a contagious disease in an apiary (B12), and implementing a biosecurity audit on a camelid‐production unit (D04) and on a horse farm (D05).

The third group of competencies exhibited an average ratio of 0.26, suggesting that around one‐quarter of participants reported insufficient confidence in these competencies. This group includes seven competencies covering different topics, such as advising owners on how to isolate an animal suspected of having a contagious and/or zoonotic disease in a camelid farm (B05), on how to isolate an animal suspected of having a contagious and/or zoonotic disease in a dog breeding facility (B09), on how to isolate an animal suspected of having a contagious and/or zoonotic disease in a kennel, a cattery, a boarding kennel, or a shelter for dogs/cats (B10), performing a risk analysis of infectious hazards pertinent to my professional practice (C01), implementing a biosecurity audit in a fish farm (D08), implementing a biosecurity audit in an apiary (D09), and identifying information sources relevant to their area of practice (E01).

The fourth group, considered of comparatively lower priority, includes 16 competencies with an average ratio of 0.14, suggesting that about 85% of participants reported confidence in these competencies.

**Figure 4:**
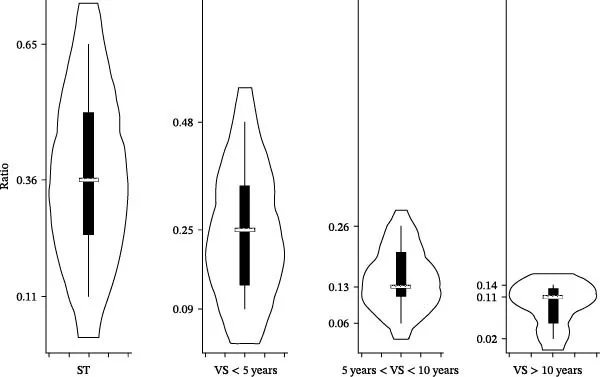
Trend of the competency ratio between the number of veterinary students and practitioners who participated in a cross‐sectional survey about biosecurity practices that partially disagree and disagree and the number of participants that partially agree and agree for 10‐biosecurity competencies. ST, final‐year veterinary students; VS, veterinary practitioners stratified by time since graduation. The 10 competencies included are competencies associated with cleaning and disinfection protocols, respectively, for surfaces (A01) and for equipment (A02); competencies related to the isolation of animal suspected of having a contagious and/or zoonotic disease in adapting a facility within a veterinary hospital or clinic (B01), in advising the owner on how to isolate a ruminant (B04), an animal in a dog breeding facility (B09), an animal in a kennel, a cattery, a boarding kennel or a shelter for dogs/cats (B10); competencies to carry out an analysis of infectious risks relevant to the field of practice (C01), to design protocols intended to protect both myself and my staff from exposure to zoonotic pathogens (C03), to communicate with owners regarding the potential hazards linked to contagious and/or zoonotic diseases affecting their animals (C04); and competency related to reference information (E01).

The influence of participant category—students and practitioners stratified by time since graduation (<5, 5–10, and >10 years)—on agreement levels for each competency was assessed when ratio information was available for over half of participants. Indeed, for the 15 competencies meeting these criteria (A01, A02, B01, B02, B04, B09, B10, C01, C02, C03, C04, C05, D01, D03, and E01), we observed an important trend: the competency ratio shows a declining gradient from students to veterinary practitioners with over 10 years of professional activity. For two additional competencies, students and early‐career veterinary practitioners (graduation <5 years ago) show nearly identical ratios, followed by a decline as time since graduation increases. Based on the 10 competencies showing a downward ratio, the decrease appears steeper during the first decade postgraduation, with a concurrent reduction in value dispersion over time (Kruskal–Wallis equality‐of‐populations rank test; *p*‐value = 0.0003). Using an ordered regression, the difference between the final‐year veterinary students (as reference) and after 5 years of graduation was not significant (*p*‐value = 0.10) but well after 5–10 years (*p*‐value = 0.001) or more than 10 years (*p*‐value  < 0.001) (Figure 4).

### 3.3. Sensitivity Analysis

**Figure 5:**
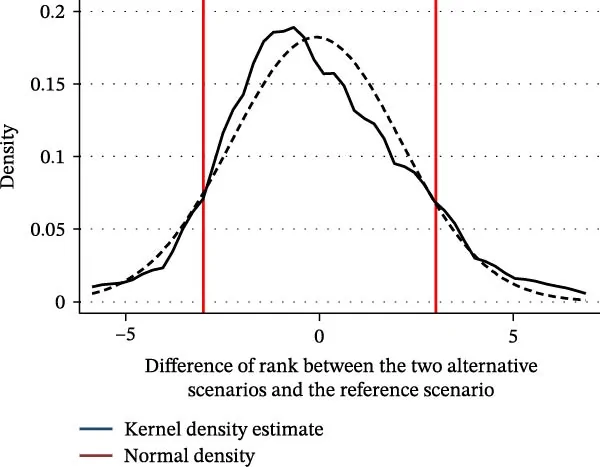
Number of competencies with change of +/− 1–6 ranks between the reference scenario (result of the current survey) and two alternative scenarios simulating first that 40% of neutral becoming with agreement and 60% becoming with disagreement, and second, the reverse. The two vertical red lines are the cut‐off fixed as more +/− 3 ranks (10% of the total number of competence ratio).

The result of the sensitivity analysis indicates that the difference of rank for the 30 competencies between each alternative scenario and the reference scenario is quite similar (two‐sample Wilcoxon rank‐sum, *p*‐value = 0.94). For this reason, we aggregate the difference in rank for the two alternative scenarios, and its distribution is normally distributed (Shapiro–Wilk test; *p*‐value = 0.68) (Figure 5). Only two competencies of alternative scenarios present more than +/− 3 ranks of difference in comparison to the reference scenario and are related to beekeeping, that is, competency to advise the owner on how to isolate a colony suspected of having a contagious disease in an apiary (B12) and to conduct a biosafety audit in an apiary (D09). Moreover, but only for the first alternative scenario, an additional competency appears and is to advise the owner on how to isolate an animal suspected of having a contagious and/or zoonotic disease in a camelid husbandry (B05). Indeed, the results of the survey can be qualified as relatively robust.

### 3.4. Expression of Training Needs

**Figure 6:**
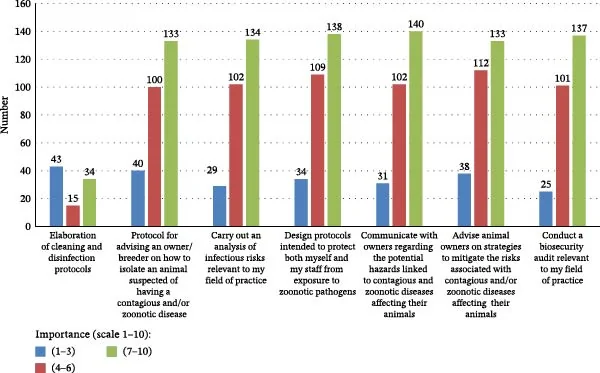
Number of veterinary students and practitioners who participated in a cross‐sectional survey about biosecurity practices indicating one or more specific training needs, stratified by importance level on a 1–10 scale.

Among the 605 participants, 276 (45.4%) expressed a need for training in one or more topics and were stratified by importance level (Figure 6). This expression is homogenous across continents (Fisher’s exact test; *p*‐value = 0.18). The level of importance for each topic was categorized as low (scores 1–3 on a 1–10 scale), moderate (scores 4–6), and high (scores 7–10). Apart from cleaning and disinfection procedures, most participants indicated either a high or moderate training need across all topics (Figure 6).

**Figure 7:**
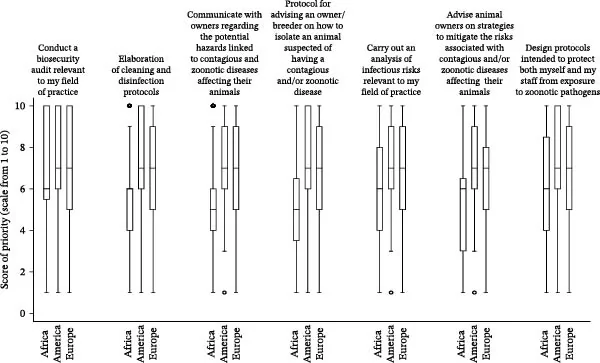
Priority scores for each competency group stratified by Africa, America, and Europe (*N* = 276 veterinary students and practitioners who participated in a cross‐sectional survey about biosecurity practices). Only data from continents with a minimum of 30 respondents are presented. The bold vertical line indicates the median, the top and bottom of each box represent, respectively, the first and third quartiles, the whiskers mark the 95% confidence‐interval limits, and small circles identify outliers.

For the next analytical step, only continents with more than 30 respondents were retained, namely, Africa (56), America (46), and Europe (162). No significant difference among the three continents was detected for the competency group related to risk analysis and biosecurity audits (Kruskal–Wallis test; *p*‐value  > 0.05). However, for all remaining competency groups, Africa exhibited significantly lower median priority scores compared with America and Europe even after Bonferroni correction (Kruskal–Wallis test; *p*‐value ≤ 0.0025) (Figure 7). Furthermore, no difference was observed between America and Europe.

## 4. Discussion

As the importance of animal biosecurity for veterinarians has increased over time, assessing biosecurity competencies and identifying associated needs will allow us to adapt and refine the training provided in this field. The results of the survey provide insight into practices reported by respondents from multiple regions and have identified several key elements for improvement. According to the sensitivity analysis performed, the results presented are relatively robust.

### 4.1. Perceived Biosecurity Competencies Associated With the Species Managed

Biosecurity audit procedures were identified as being particularly important for traditional and nontraditional pets, such as horses, camelids, fish, and bees. These procedures should be introduced or reinforced. While several publications indexed in PubMed address livestock biosecurity audits at the farm level [12–14], far fewer are available for veterinary education establishments [5, 15], for companion animals, [16], horses [17, 18], camelids [19], fish [20], and bees [21, 22]. Reports of less‐than‐optimal biosecurity audit methods are available as well [23]. Nonetheless, implementing biosecurity checklists supports the progressive uptake of good practices among farmers and warrants broader promotion [24]. More resources should be allocated to expanding biosecurity audit checklists applicable to a wider diversity of species.

Other competencies requiring reinforcement include animal isolation procedures, that is, the segregation of sick animals from healthy individuals. Although few scientific papers have been dedicated to animal isolation [25], this topic has recently been identified as a concern in livestock [26]. Evidence‐based, species‐specific operational protocols are required to improve biosecurity at the farm level.

Risk analysis—specifically the assessment of infectious hazards relevant to the veterinarians’ field of practice—is another competency requiring further development. Both qualitative [27] and quantitative approaches to risk‐introduction analysis [28–32] are available—typically applied at national or border control levels—yet they are rarely integrated into routine professional practice. Moreover, only a limited number of studies address these issues at the farm level or along the broader value chain [33–37]. The broad dissemination of risk analysis competencies is required across diverse animal species and operational contexts. This could enhance our understanding of how individual biosecurity measures, as well as their combined implementation, contribute to risk mitigation.

Finally, the ability to access relevant and validated biosecurity pertinent to veterinarians’ field of practice is essential. The dissemination of information on animal biosecurity constitutes a core component of the FAO’s Progressive Management Pathway for Terrestrial Animal Biosecurity [38]. Some standard operating procedures (SOP) for animal biosecurity [5] and dedicated knowledge centers on‐farm‐level biosecurity are available (e.g., https://www.cfsph.iastate.edu/biosecurity/), but their dissemination remains insufficient. The creation of the WABA represents a positive step toward the progressive implementation of biosecurity recommendations. The monthly webinars on animal biosecurity, jointly organized by the Food and Agriculture Organization of the United Nations (FAO) and the Scientific and Information Committee of the WABA, constitute an effective model for structured information sharing (https://www.fao.org/animal-health/resources/webinars/en/).

Furthermore, when comparing final‐year students with practitioners who graduated <5 years ago, 5–10 years ago, or >10 years ago, we identified a noteworthy pattern. A marked decline was observed in both the ratio of competencies considered necessary as time since graduation increased up to 10 years and in the dispersion of these competency scores. However, after 10 years from the graduation, a slow decrease of the ratio and dispersion was observed. This finding appears to reflect a perceived increase in the time required to acquire the targeted competencies. It is well established that newly graduated veterinarians commonly experience “knowledge decay”—the progressive loss of detailed, nonapplied theoretical information—while concurrently developing practical “competencies,” that is, real‐world, hands‐on clinical skills. The transition from a textbook‐oriented mindset to practice‐ready autonomy is achieved primarily through structured mentorship—often guided by the 4Rs framework (reflection, recommendations, resources, and referrals)—supported by targeted continuing professional development and repeated practical application [39].

### 4.2. Prioritizing Training Requirements

Except for cleaning and disinfection protocols, topics such as animal isolation, risk analysis, biosecurity auditing, and information sourcing were more frequently rated as of high or moderate importance than as of low importance by most participants. These results underscore the need to integrate all these topics into practical biosecurity training programs for veterinarians.

Apart from risk analysis and biosecurity audits, the median priority score for cleaning and disinfection procedures, animal isolation protocols, and information sourcing was significantly lower in Africa than in America and Europe. As noted by Pinto Jimenez et al. [40], cleaning and disinfection procedures support the adoption of hygienic practices, the implementation of standardized protocols, and the enforcement of policies that promote adequate hygiene within households, among individuals, and in areas where animals are kept. This contributes to preventing the introduction and dissemination of pathogens among humans, animals, and the environment. However, in Africa, the implementation of cleaning and disinfection procedures within animal biosecurity systems is more challenging due to widespread resource constraints, predominantly informal and communal livestock farming systems, and harsh environmental conditions. These combined factors make the consistent application of SOPs in Africa particularly difficult. Implementing biosecurity measures in rural settings is considerably more complex than policy frameworks and formal recommendations typically imply. This may be constrained by limited resources, weaknesses in disease‐prevention and control policies, insufficient enforcement, sociocultural determinants—including gender and equity considerations—and conflicts with local cultural practices [41–44]. Further studies—particularly action research approaches—are therefore required to investigate the determinants of biosecurity adoption among community members living in rural areas, with the aim of identifying actionable leverages at the community level [45, 46]. To address these specific regional challenges, targeted capacity‐building initiatives are currently playing a pivotal role. For instance, the Regional Training and Certification Program (RTCP) launched by the Africa Centres for Disease Control and Prevention (Africa CDC) targets professionals across both public health and animal health sectors, actively promoting an operational One Health approach [47]. Additionally, international collaborations—such as the establishment of regional trainer networks in North Africa by Sandia National Laboratories and the Defense Threat Reduction Agency (DTRA)—are delivering regular, localized biosafety and biosecurity training. Such programs are essential for harmonizing Biorisk Management competencies, providing tailored mentorship, and building a sustainable network of experts capable of implementing SOPs in resource‐limited settings. However, while these initiatives represent a massive step forward, they are currently heavily focused on laboratory biosafety and biosecurity. The next major challenge will be to extend this level of standardized training to field applications and farm biosecurity, which perfectly aligns with the educational gaps identified in this study.

Isolating animals also represents a major biosecurity challenge in Africa due to the predominance of smallholder farming systems, the use of shared communal grazing areas, porous borders, and limited veterinary infrastructure. These constraints facilitate the rapid dissemination of high‐impact transboundary animal diseases (TAD) such as African swine fever (ASF) and foot‐and‐mouth disease (FMD).

Although several websites provide biosecurity‐related information, substantial room for improvement remains—particularly in incorporating guidance on how to communicate about biosecurity, analyses of its costs and benefits, self‐assessment tools, and visual materials such as videos and illustrations describing biosecurity measures [48]. A recent African study showed that farmers with access to information on biosecurity measures were 25% more likely to adopt a greater number of biosecurity practices [49]. Indeed, access to information constitutes the primary driver of biosecurity adoption.

More broadly, a key remaining challenge is the development of biosecurity SOPs that are tailored to resource‐limited settings and feasible to implement at the community level. From a One Health perspective, the biosecurity competency gaps identified in this survey have direct implications for TAD preparedness. Veterinarians operate at the critical human–animal interface where zoonotic pathogens emerge and spread [50]. Deficiencies in risk analysis, biosecurity audit, and animal isolation—the three competency domains most in need of improvement—are precisely the competencies required for early detection and containment of TAD, such as highly pathogenic avian influenza, ASF, and Rift Valley fever. In sub‐Saharan Africa, where the One Health landscape is characterized by high livestock–human contact and limited veterinary infrastructure [51], the lower priority scores for biosecurity training observed in our survey are of particular concern. Recent studies confirm that veterinary students in the Global South demonstrate poor knowledge of infection control and biosecurity measures despite awareness of zoonosis risks [52]. Furthermore, understanding the behavioral determinants of biosecurity adoption, such as capability, opportunity, and motivation [53], is essential for designing effective training interventions. Strengthening biosecurity competencies through targeted, modular training programs [8] would therefore contribute not only to animal health but also to global health security and pandemic preparedness, in direct alignment with the FAO Progressive Management Pathway for Terrestrial Animal Biosecurity [38] and the COHFE One Health framework [6].

The main strengths of this survey are its coverage across 66 countries and the balanced representation of veterinary students and practitioners with varying levels of professional experience. However, several limitations should be acknowledged. The snowball sampling strategy, employed in the absence of a formal sampling frame, may have introduced selection bias (overrepresentation of individuals with prior interest in biosecurity), resulting in an overrepresentation of European and African respondents. Furthermore, the competency assessment relied on self‐reported perceptions rather than objective measurement, which may be subject to social desirability bias. The cross‐sectional design also precludes causal inference regarding the observed association between years of experience and competency levels.

## 5. Conclusion

The results provide insight into practices reported by respondents from multiple countries. This survey highlighted several domains of biosecurity training in which significant improvements are required. Identifying these competencies and needs in relation to animal biosecurity will enable the adaptation of training programs, thereby contributing to the achievement of the 3rd and 10th Sustainable Development Goals—promoting good health and well‐being and reducing inequalities within and between countries, respectively.

## Author Contributions

Claude Saegerman and Marion Allano designed the survey. Véronique Renault transcribed the survey in KoboToolbox. Claude Saegerman analyzed the data, interpreted the results, and wrote the manuscript. Blerta Mehmedi, Jorge Ron‐Román, Amel Adel, Bachir Souley‐Kouato, A. K. M. Anisur Rahman, and Mari‐France Humblet pretested and refined the questionnaire. Claude Saegerman and Marion Allano edited the final version.

## Funding

This study was supported by the University of Liege.

## Disclosure

All the authors have reviewed the manuscript.

## Ethics Statement

A cover letter and a questionnaire were submitted to and approved by the data privacy officer of the University of Liège to verify compliance with the General Data Protection Regulation. The survey was then approved by the Ethics Committee for the Humanities and Social Sciences at the University of Liège (Reference Number 2025−50). Informed consent was obtained from all participants prior to their inclusion in the survey.

## Conflicts of Interest

The authors declare no conflicts of interest.

## Supporting Information

Additional supporting information can be found online in the Supporting Information section.

## Supporting information


**Supporting Information 1** Questionnaire S1: Questionnaire survey.

**Supporting Information 2** Figure S1: Groups of species identified through a regression tree analysis.

**Supporting Information 3** Table S1: Number of veterinary students and practitioners with a specific level of agreement regarding perceived biosecurity competencies related to the species managed in a cross‐sectional survey about biosecurity practices, 2026.

## Data Availability

The primary dataset used in this study is publicly available, and additional data may be obtained upon reasonable request from the corresponding author.
