# Supplementary material for: Biosecurity Competencies and Educational Needs Among Veterinary Professionals: A Multinational Cross‐Sectional Survey
Source: Transbound Emerg Dis. 2026 Sep 15;2026:7275694. doi: 10.1155/tbed/7275694 (PMC13575878; doi:10.1155/tbed/7275694)
Supplement: Supplementary file 1 [file TBED-2026-7275694-s001.docx]

**Education needs in applied veterinary biosecurity**

**Anonymous questionnaire for final-year veterinary students and veterinary practitioners**

Currently, veterinary biosecurity is of increasing importance and has been progressively integrated into the veterinary medicine curriculum as well as into the European Animal Health Law (Regulation (EU) 2016/429). A new chapter of the Terrestrial Animal Health Code on biosecurity is currently being prepared by the World Organisation for Animal Health, and the World Animal Biosecurity Association (WABA) will be officially created this year.

However, in view of these developments, no survey has yet been conducted to assess the educational needs of veterinary students and practitioners in the field of applied animal biosecurity.

This survey aims to address this gap. Identifying these needs will enable the development of targeted educational interventions. The data collected in this online survey will not allow you to be identified, either directly or indirectly. Data will be collected and analysed by the Research Unit of Epidemiology, Risk Analysis and Biosecurity Applied to Veterinary Sciences of the University of Liège. They may be shared for research purposes.

If you have any concerns about this study or how the data will be processed, please contact Claude Saegerman in the first instance at: claude.saegerman@uliege.be. At the end of the study, the University of Liège will send a summary report to veterinary practitioners.

We would like to thank all final-year veterinary students and veterinary practitioners who contribute to a better understanding of veterinary biosecurity educational needs.

Yours sincerely, Prof. Claude Saegerman.

**Section 1. Participant’s profile**

**Q01. I am (Answer required. Single choice.)**

o A student in final year of veterinary studies

o Veterinary surgeon graduated less than 5 years ago

o Veterinary surgeon graduated between 5 and 10 years ago

o Veterinary surgeon graduated more than 10 years ago

o Other, please specify ...............................

**Q02. I work (check all that apply to your situation) (Answer required. Multiple choice.)**

o In a veterinary clinic

o In ambulatory practice

o In an academic environment

o In a government body (e.g. Ministry, Agency, Association)

o In the pharmaceutical industry

o In a non-governmental organization (e.g. VSF)

o Other, please specify ...............................

**Q03. What animal species do you work with? (check all that apply to your situation) (Answer required. Multiple choice).**

o Dogs, cats, ferrets

o Exotic/non-conventional pets (reptiles, birds, small mammals, etc.)

o Equids

o Cattle

o Small ruminants

o Camelids

o Pigs

o Poultry

o Fishes

o Bees

o Wildlife (e.g. shelter / rehabilitation centres)

o Not applicable to my area of practice

o Other, please specify ...............................

**Section 2. Competency Self-Assessment**

**Q04. Please choose the option that best describes your opinion on the following statements: (Answer required. Likert. Condition the sub-answers on the answers to QO3).**

|  | Does not apply to my practice | Disagree | Partially disagree | Neutral | Partially agree | Agree |
| --- | --- | --- | --- | --- | --- | --- |
| I feel competent in developing cleaning/disinfection protocols for the **surfaces** (e.g. consultation table) I use (choice of product type, procedure). |  |  |  |  |  |  |
| I feel competent in developing cleaning/disinfection protocols for the **equipment** I use (choice of product type, procedure). |  |  |  |  |  |  |
| I feel competent in adapting a premise, in a veterinary hospital or clinic*, to isolate an animal suspected of a contagious and/or zoonotic disease.  **A veterinary hospital is defined as a veterinary facility whose infrastructure includes at least one area dedicated to the hospitalization of more than one patient.* |  |  |  |  |  |  |
| I feel competent to recommend to the owner how to isolate a **dog, cat or ferret** suspected of a contagious and/or zoonotic disease. |  |  |  |  |  |  |
| I feel competent to recommend to the owner how to isolate an **exotic/non-conventional pet** (reptile, bird, small mammal, etc.) suspected of a contagious and/or zoonotic disease. |  |  |  |  |  |  |
| I feel competent to recommend to the owner how to isolate an animal suspected of a contagious and/or zoonotic disease in a farm of ruminants or camelids. |  |  |  |  |  |  |
| I feel competent to recommend to the farmer how to isolate an animal (pig pen) suspected of a contagious and/or zoonotic disease in **a pig farm**. |  |  |  |  |  |  |
| I feel competent to recommend to an owner how to isolate an animal (poultry pen) suspected of a contagious and/or zoonotic disease in a **poultry farm**. |  |  |  |  |  |  |
| I feel competent to recommend to the owner how to isolate an animal suspected of a contagious and/or zoonotic disease in a **horse stable**. |  |  |  |  |  |  |
| I feel competent to recommend to the owner how to isolate an animal suspected of a contagious and/or zoonotic disease in a **dog breeding facility**. |  |  |  |  |  |  |
| I feel competent to recommend to an owner how to isolate an animal suspected of having a contagious and/or zoonotic disease in a **cattery**. |  |  |  |  |  |  |
| I feel competent to recommend how to isolate an animal suspected of a contagious and/or zoonotic disease in a boarding **kennel or a shelter** for dogs/cats. |  |  |  |  |  |  |
| I feel competent to recommend to the owner how to isolate a batch of animals suspected of a contagious and/or zoonotic disease in a **fish farm**. |  |  |  |  |  |  |
| I feel competent to recommend to the owner how to isolate a colony suspected of a contagious disease in an **apiary**. |  |  |  |  |  |  |
| I feel competent to recommend to the owner of a **shelter/wildlife rehabilitation centre** how to isolate an animal suspected of having a contagious disease. |  |  |  |  |  |  |
| I feel competent to conduct an analysis of infectious risks in relation to my area of practice. |  |  |  |  |  |  |
| I feel competent to develop protocols to reduce the risk of contagion between patients (in a clinic). |  |  |  |  |  |  |
| I feel competent to develop protocols to reduce the risk of zoonotic transmission for myself and the staff for whom I am responsible. |  |  |  |  |  |  |
| I feel competent to discuss with owners the risks associated with contagious and/or zoonotic diseases in their animal(s). |  |  |  |  |  |  |
| I feel competent to advise animal owners on strategies for mitigating the risks associated with contagious and/or zoonotic diseases in their animal(s). |  |  |  |  |  |  |
| I feel competent to conduct a biosecurity audit in a **community of** dogs, cats or in a ferret farm). |  |  |  |  |  |  |
| I feel competent to conduct a biosecurity audit at a **seller of exotic/non-conventional pets** (e.g. reptiles, exotic birds). |  |  |  |  |  |  |
| I feel competent to conduct a biosecurity audit in a **ruminant farm**. |  |  |  |  |  |  |
| I feel competent to conduct a biosecurity audit in a **horse stable**. |  |  |  |  |  |  |
| I feel competent to conduct a biosecurity audit in a **pig farm**. |  |  |  |  |  |  |
| I feel competent to conduct a biosecurity audit in a **poultry farm**. |  |  |  |  |  |  |
| I feel competent to conduct a biosecurity audit in a **fish farm**. |  |  |  |  |  |  |
| I feel competent to conduct a biosafety audit in a **beekeeping farm**. |  |  |  |  |  |  |
| If necessary, I know where to find **reference information** related to my area of practice. |  |  |  |  |  |  |

**Q05. Please give some details related to your previous answers, if necessary (max 250 words).**

Multiline text - max 250 words.

Enter your answer

**Q06. Which subject(s) do you think should be added to the curriculum of veterinary students concerning the operational aspect of infection prevention and control in veterinary medicine*?**

Multiline text - max 250

words.

**The question is mainly directed to veterinary practices/clinics and outpatient clinics.*

Enter your answer

**Section 3. Additional educational needs**

**Q07. Can you identify any additional educational needs in relation to the points covered in the questionnaire?**

o NO

o YES

**Q08. If YES to question Q07, indicate your priorities on a scale from 0 (corresponding to no need) to 10 (corresponding to a very important need) for each of the following sections:**

| Section | Priority: scale from 1 (minimum) to 10 (maximum) |
| --- | --- |
| Develop cleaning/disinfection protocols |  |
| Protocol for recommending to an owner/breeder how to isolate an animal suspected of a contagious and/or zoonotic disease |  |
| Conduct an analysis of infectious risks related to my area of practice |  |
| Develop protocols to reduce the risks of zoonosis transmission for myself and the staff for whom I am responsible |  |
| Discuss with owners the risks associated with contagious and zoonotic diseases in their animal(s) |  |
| Advise owners on strategies to mitigate the risks associated with contagious and/or zoonotic diseases in their animal(s). |  |
| Conduct a biosecurity audit related to my area of practice |  |

**Thank you very much for your appreciated contribution.**
