# Supplementary material for: Biosecurity Competencies and Educational Needs Among Veterinary Professionals: A Multinational Cross‐Sectional Survey
Source: Transbound Emerg Dis. 2026 Sep 15;2026:7275694. doi: 10.1155/tbed/7275694 (PMC13575878; doi:10.1155/tbed/7275694)
Supplement: Supplementary file 2 [file TBED-2026-7275694-s002.docx]

**Appendix B.** Groups of species identified through a regression tree analysis

Legend: Avr, Average; STD, Standard deviation; N, number of participants.
