# Supplementary material for: Biosecurity Competencies and Educational Needs Among Veterinary Professionals: A Multinational Cross‐Sectional Survey
Source: Transbound Emerg Dis. 2026 Sep 15;2026:7275694. doi: 10.1155/tbed/7275694 (PMC13575878; doi:10.1155/tbed/7275694)
Supplement: Supplementary file 3 [file TBED-2026-7275694-s003.docx]

**Table S1.** Number of veterinary students and practitioners with a specific level of agreement regarding perceived biosecurity competencies related to the species managed in a cross-sectional survey about biosecurity practices, 2026

| Competency by thematic area | Number of participants | | | | |  |  |
| --- | --- | --- | --- | --- | --- | --- | --- |
|  | Disagree (D) | Partially disagree (PD) | Neutral (N) | Partially agree (PA) | Agree (A) | Ratio = [(PD)+(D)]/[(A)+(PA)] |  |
| *[A] - Cleaning and disinfection protocols* | | | | | | | |
| A01. In developing cleaning/disinfection protocols for surfaces (e.g. consultation tables), including the choice of product type and procedure | 21 | 65 | 56 | 207 | 254 | 0.19 |  |
| A02. In developing cleaning/disinfection protocols for the equipment, including the choice of product type and procedure | 31 | 68 | 59 | 209 | 258 | 0.21 |  |
| Sub-total number [A] | 52 | 133 | 115 | 416 | 512 | 0.20 |  |
| Sub-total percentage [A] | 4.2 | 10.8 | 9.4 | 33.9 | 41.7 | - |  |
| *[B] - Animal isolation* | | | | | | | |
| B01. In adapting a facility within a veterinary hospital or clinic to isolate an animal suspected of having a contagious and/or zoonotic disease | 20 | 35 | 35 | 166 | 203 | 0.15 |  |
| B02. To advise the owner on how to isolate a dog, cat or ferret suspected of having a contagious and/or zoonotic disease | 17 | 38 | 26 | 113 | 160 | 0.20 |  |
| B03. To advise the owner on how to isolate an exotic/non-conventional pet (reptile, bird, small mammal, etc) suspected of having a contagious and/or zoonotic disease | 10 | 10 | 9 | 21 | 30 | 0.39 |  |
| B04. To advise the owner on how to isolate an animal suspected of having a contagious and/or zoonotic disease in a ruminant husbandry | 10 | 23 | 22 | 116 | 201 | 0.10 |  |
| B05. To advise the owner on how to isolate an animal suspected of having a contagious and/or zoonotic disease in a camelid husbandry | 5 | 5 | 10 | 7 | 26 | 0.30 |  |
| B06. To advise the farmer on how to isolate an animal (pig pen) suspected of having a contagious and/or zoonotic disease in a pig farm | 3 | 10 | 16 | 48 | 93 | 0.09 |  |
| B07. To advise the owner on how to isolate an animal (poultry pen) suspected of having a contagious and/or zoonotic disease in a poultry farm | 5 | 12 | 20 | 34 | 100 | 0.13 |  |
| B08. To advise the owner on how to isolate an animal suspected of having a contagious and/or zoonotic disease in a equine husbandry | 13 | 13 | 14 | 76 | 83 | 0.16 |  |
| B09. To advise the owner on how to isolate an animal suspected of having a contagious and/or zoonotic disease in a dog breeding facility | 27 | 34 | 35 | 119 | 133 | 0.24 |  |
| B10. To advise the owner on how to isolate an animal suspected of having a contagious and/or zoonotic disease in a kennel, a cattery, a boarding kennel or a shelter for dogs/cats | 20 | 42 | 46 | 120 | 117 | 0.26 |  |
| B11. To advise the owner on how to isolate a batch of animals suspected of having a contagious and/or zoonotic disease in a fish farm | 7 | 2 | 4 | 6 | 20 | 0.35 |  |
| B12. To advise the owner on how to isolate a colony suspected of having a contagious disease in an apiary | 4 | 4 | 0 | 8 | 13 | 0.38 |  |
| B13. To advise the owner of a shelter/wildlife rehabilitation centre on how to isolate an animal suspected of having a contagious and/or zoonotic disease | 2 | 2 | 3 | 12 | 24 | 0.11 |  |
| Sub-total number [B] | 143 | 230 | 240 | 846 | 1203 | 0.18 |  |
| Sub-total percentage [B] | 5.4 | 8.6 | 9.0 | 31.8 | 45.2 | - |  |
| *[C] - Risk analysis* | | | | | | | |
| C01. To carry out an analysis of infectious risks relevant to my field of practice | 49 | 67 | 83 | 222 | 218 | 0.26 |  |
| C02. To design procedures aimed at reducing the risk of contagion between patients within a veterinary clinic. | 9 | 24 | 43 | 180 | 157 | 0.10 |  |
| C03. To design protocols intended to protect both myself and my staff from exposure to zoonotic pathogens | 12 | 39 | 54 | 247 | 288 | 0.10 |  |
| C04. To communicate with owners regarding the potential hazards linked to contagious and/or zoonotic diseases affecting their animal(s) | 7 | 23 | 42 | 222 | 348 | 0.05 |  |
| C05. To advise animal owners on strategies to mitigate the risks associated with contagious and/or zoonotic diseases affecting their animal(s) | 6 | 31 | 70 | 235 | 301 | 0.07 |  |
| Sub-total number [C] | 83 | 184 | 292 | 1106 | 1312 | 0.11 |  |
| Sub-total percentage [C] | 2.8 | 6.2 | 9.8 | 37.2 | 44.1 | - |  |
| *[D] - Biosecurity audit* | | | | | | | |
| D01. To conduct a biosecurity audit in a community of dogs or cats, or in a ferret-breeding facility) | 64 | 49 | 64 | 93 | 68 | 0.70 |  |
| D02. To conduct a biosecurity audit at a seller of exotic/non-conventional pets (eg reptiles, exotic birds) | 23 | 12 | 10 | 17 | 15 | 1.09 |  |
| D03. To conduct a biosecurity audit in a ruminant husbandry | 26 | 26 | 58 | 125 | 133 | 0.20 |  |
| D04. To conduct a biosecurity audit in a camelid husbandry | 9 | 2 | 11 | 12 | 18 | 0.37 |  |
| D05. To conduct a biosecurity audit in a horse farm | 30 | 20 | 28 | 61 | 58 | 0.42 |  |
| D06. To conduct a biosecurity audit in a pig farm | 11 | 13 | 21 | 51 | 72 | 0.20 |  |
| D07. To conduct a biosecurity audit in a poultry farm | 9 | 9 | 20 | 39 | 94 | 0.14 |  |
| D08. To conduct a biosecurity audit in a fish farm | 7 | 1 | 4 | 13 | 14 | 0.30 |  |
| D09. To conduct a biosecurity audit in an apiary | 2 | 2 | 7 | 6 | 11 | 0.24 |  |
| Sub-total number [D] | 181 | 134 | 223 | 417 | 483 | 0.35 |  |
| Sub-total percentage [D] | 12.6 | 9.3 | 15.5 | 29.0 | 33.6 | - |  |
| *[E] – Information sourcing* | | | | | | | |
| E01. To identify appropriate sources of information relevant to my field of practice | 51 | 57 | 79 | 187 | 274 | 0.23 |  |
| Percentage [E] | 7.9 | 8.8 | 12.2 | 28.9 | 42.3 | - |  |
| Total number ([A]+[B]+[C]+[D]+[E]) | 459 | 681 | 870 | 2785 | 3510 | 0.18 |  |
| Total percentage ([A]+[B]+[C]+[D]+[E]) | 5.5 | 8.2 | 10.5 | 33.5 | 42.3 | - |  |
